# Supplementary material for: Bisdemethoxycurcumin Alleviates Dextran Sodium Sulfate-Induced Colitis via Inhibiting NLRP3 Inflammasome Activation and Modulating the Gut Microbiota in Mice
Source: Antioxidants (Basel). 2022 Oct 7;11(10):1994. doi: 10.3390/antiox11101994 (PMC9598232; doi:10.3390/antiox11101994)
Supplement: Supplementary file 1 [file antioxidants-11-01994-s001.zip › antioxidants-1906050-supplementary.pdf]

antioxidants

MDPI

Supplementary Materials

Bisdemethoxycurcumin Alleviates Dextran Sodium Sulfate-induced Colitis via Inhibiting NLRP3 Inflammasome Activation and Modulating the Gut Microbiota in Mice

Jingfei Zhang, Qiming Li, Xin Zhang, Yanan Chen, Yufang Lu, Xinyu Wang, Lili Zhang and Tian Wang

Table S1. The disease activity index scoring system.

Score	Weight Loss	Stool Consistency	Blood Stools
0	<1 %	normal	normal
1	1-5 %	-	-
2	5-10 %	loose stool	slight
3	10-15 %	-	-
4	>15 %	diarrhea	gross bleeding

Table S2. Sequences of primers used for RT–PCR.

Genes		Primer Sequence	Accession	Product (bp)
NLRP3	Forward	CTCGTCACCATGGGTTCTGGT	NM_145827.4	230
	Reverse	AACGGACACTCGTCATCTTCA		
ASC	Forward	TGAGCAGCTGCAAACGACTA	NM_023258.4	200
	Reverse	CACGAACTGCCTGGTACTGT		
IL-1β	Forward	TGCCACCTTTTGACAGTGATG	NM_008361.4	220
	Reverse	AAGGTCCACGGGAAAGACAC		
IL-18	Forward	CCTTTGAGGCATCCAGGACAA	NM_008360.2	209
	Reverse	CGGGGCCTGAGGATTATAGC		
GSDMD	Forward	GATCAAGGAGGTAAGCGGCA	NM_026960.4	195
	Reverse	CACTCCGGTTCTGGTTCTGG		
ZO-1	Forward	CTCTCCTGTACCTCTTGAGCC	NM_009386.2	263
	Reverse	CAGAAATCGTGCTGATGTGCC		
Occludin	Forward	CCGGCCGCCAAGGTTC	NM_008756.2	78
	Reverse	GCTGATGTCACTGGTCACCTA		
Claudin-1	Forward	TATGACCCCTTGACCCCCAT	NM_016674.4	132
	Reverse	AGAGGTTGTTTTCCGGGGAC		
Bax	Forward	CACTAAAGTGCCCGAGCTGA	NM_007527.3	84
	Reverse	TCTTGGATCCAGACAAGCAGC		
Bcl2	Forward	GAACTGGGGGAGGATTGTGG	NM_009741.5	194
	Reverse	GCATGCTGGGGCCATATAGT		
Caspase 1	Forward	TGGGACCCTCAAGTTTTGCC	XM_040690807.2	490
	Reverse	GCTCCAACCCTCGGAGAAAG		
Caspase 3	Forward	GAGCTTGGAACGGTACGCTA	NM_001284409.1	234
	Reverse	CCGTACCAGAGCGAGATGAC		
Caspase 7	Forward	GCCTCTGGGACTTTTGCTTTC	NM_007611.2	189
	Reverse	ATAGAGGAGCGGTCTGGCTT		
Caspase 9	Forward	AAAGTGGCTCCTGGTACATCG	NM_015733.5	113
	Reverse	CCCTTTCGCAGAAACAGCAT		
β-actin	Forward	TGTACCCAGGCATTGCTGAC	NM_007393.5	238
	Reverse	AACGCAGCTCAGTAACAGTCC		
GAPDH	Forward	TCTCCTGCGACTTCAACA	NM_001289726.1	117
	Reverse	TGTAGCCGTATTCATTGTCA		

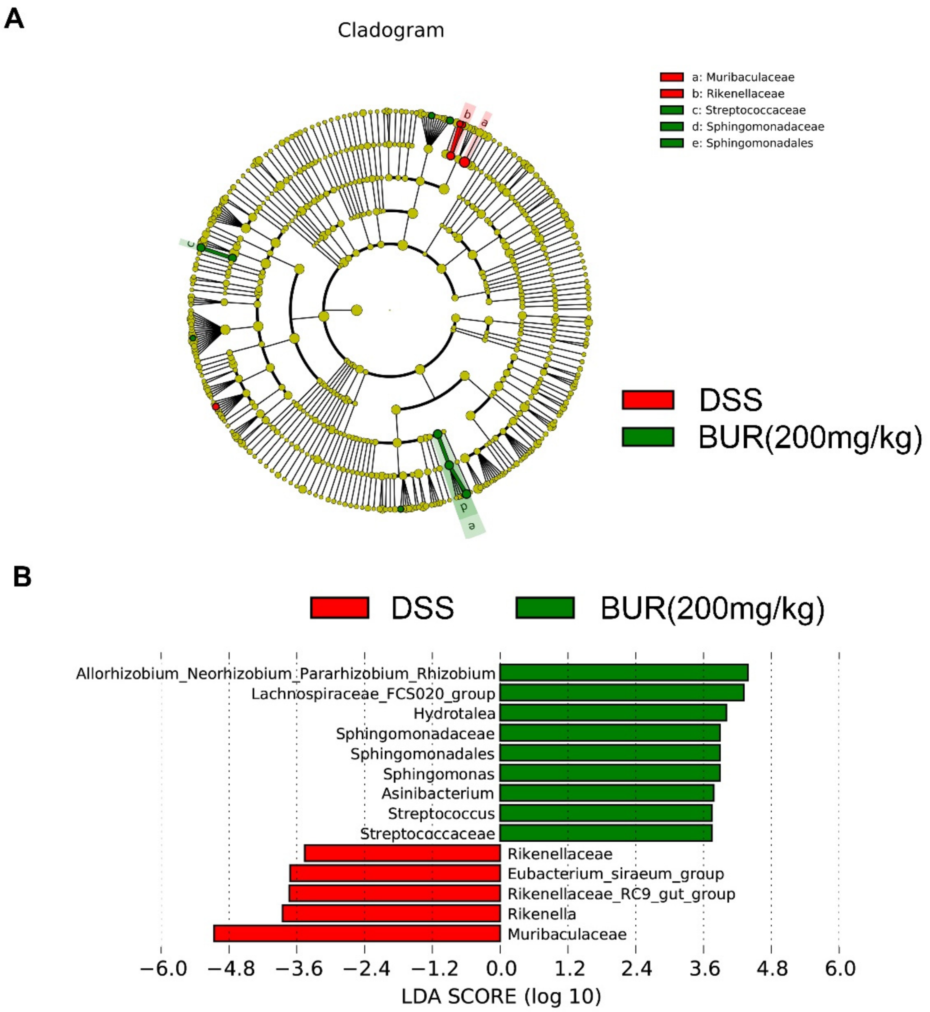

Figure S1. LEfSe analysis of the gut microbial composition among treatment groups (LDA > 2, $P < 0.05$). (**A**) Cladogram representation of the microbiota taxa between the DSS group and BUR (200 mg/kg) group. (**B**) LDA of the microbiota taxa between the DSS group and BUR (200 mg/kg) group.
